# Erratum for Euro Surveill. 2018;23(21)

**DOI:** 10.2807/1560-7917.ES.2018.23.22.180531-2

**Published:** 2018-05-31

**Authors:** Eurosurveillance editorial team

**Affiliations:** 1 European Centre for Disease Prevention and Control (ECDC), Stockholm, Sweden

In the article ‘Hepatitis A: an epidemiological survey in blood donors, France 2015 to 2017’, published on 24 May 2018, the year in some cells of the Table was incorrectly displayed. The mistake was corrected on 25 May 2018.
